# Advanced stratification analyses in molecular association meta-analysis: methodology and application

**DOI:** 10.1186/s12874-020-01020-z

**Published:** 2020-06-08

**Authors:** Shuhuang Lin, Yukun Ma, Zunnan Huang

**Affiliations:** 1 Key Laboratory of Big Data Mining and Precision Drug Design of Guangdong Medical University, Research Platform Service Management Center, Guangdong Medical University, Dongguan, 523808 Guangdong China; 2 The Second School of Clinical Medicine, Guangdong Medical University, Dongguan, 523808 Guangdong China; 3 Institute of Marine Biomedical Research, Guangdong Medical University, Zhanjiang, 524023 Guangdong China

**Keywords:** Stratified data, Genetic polymorphisms, Biomarkers, Risk factors, Mixed-effects model, Comprehensive meta-analysis

## Abstract

**Background:**

Stratification analyses have been widely utilized in molecular association meta-analyses to estimate the interaction between genetic and environmental factors or to control for the confounding variables linked to a disease. Two calculation methods utilized in practical research, which are known as the variants of factorial stratification analysis and confounder-controlling stratification analysis in our nomenclature, have been applied in previous studies, but none of which have presented a methodology and application for these analyses.

**Methods:**

In this paper, these two approaches are integrated and further developed into a standard procedure for stratification analysis. We first propose the advanced statistical methodology and theoretical algorithm of these three types of stratification analysis and then provide two example applications in meta-analyses of molecular association to illustrate the computing processes and interpretation of the results.

**Results:**

The standard stratification analysis synthesizes the advantages of the first two practical methods, including identifying and controlling confounding moderators or revealing and calculating gene-environment interactions, to efficiently classify the real influence of various investigated factors on a disease in the general population. Additional challenges concerning this method and their potential solutions are also discussed, such as the approach to utilizing only the partially stratified data available in meta-research practice.

**Conclusions:**

The standard stratification method will be extensively applicable to rapidly expanding future research on the complex relationships among genetics, environment, disease, and other variables.

## Background

**Fig. 1:**
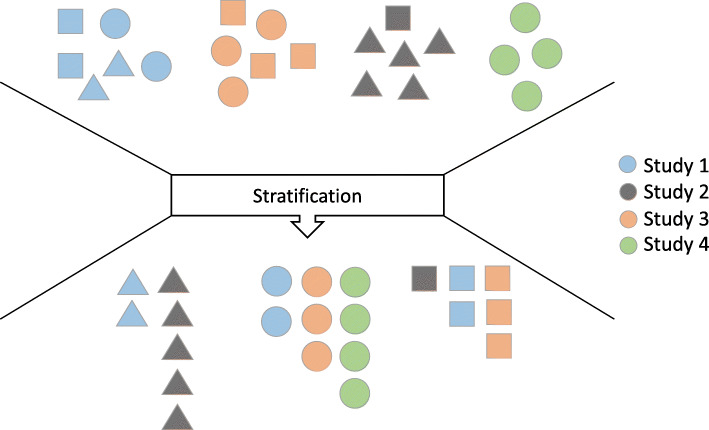
Schematic of the stratification in meta-analyses

In molecular association meta-analysis, stratification analysis (also called stratified analysis [1] or risk-stratification analysis [2]) is frequently utilized to compare the size of the effects of a genetic or epigenetic factor among the studied population with variants of a characteristic, to control this confounder in clarifying the real effects of the genetic factor, or to reveal the interaction or effect modification occurring between the genetic susceptibility and another exposure. The term “stratification” denotes that an overall study population is separated into several strata based on characteristics (e.g., smoking status [3] or drug intake status [4]) that may have an influence on the clinical indexes (e.g., a dichotomous disease outcome, such as the development of cancer or not) (shown in Fig. 1). Stratified data are generally classified as in Table 1.

**Table 1:** Sorting table for stratified data in molecular association meta-analysis of case-control studies

No. of studies	Genetic susceptibility	Stratum_1_		Stratum_2_		…	Stratum_q_	
		Cases	Controls	Cases	Controls		Cases	Controls
1	+	a_11_	b_11_	a_12_	b_12_	…	a_1q_	b_1q_
	–	c_11_	d_11_	c_12_	d_12_		c_1q_	d_1q_
2	+	a_21_	b_21_	a_22_	b_22_	…	a_2q_	b_2q_
	–	c_21_	d_21_	c_22_	d_22_		c_2q_	d_2q_
…								
k	+	a_k1_	b_k1_	a_k2_	b_k2_	…	a_kq_	b_kq_
	–	c_k1_	d_k1_	c_k2_	d_k2_		c_kq_	d_kq_

Although stratification analysis is powerful in solving heterogeneity problems and occasionally provides accurate or reasonable estimations and has been widely applied in meta-analyses of observational or experimental studies of inheritance or clinical intervention [2–10], we noted three methodological deficiencies of its application in previous molecular association meta-analyses:

- Confusion of effect model selection to synthesize original data in a meta-analytic stratification analysis. Our previous study pointed out that, in some meta-analyses, stratified data from various studies were merely added together as in a primary case-control study and were pooled for a crude size of effect without considering the heterogeneity across each independent study [2, 5, 6]. On the other hand, for synthesizing data across strata within original studies, the data from different strata are sometimes pooled using a fixed-effect model [11, 12] or a random-effects model [13, 14].
- Failure to control confounding factors and to reveal a true effect of inheritance among the studied population. Numerous previously reported meta-analyses have detected the effect of genetic variants among subpopulations with a particular characteristic rather than the overall study population with a larger sample size [4, 7–10].
- A lack of homogeneity testing across strata and quantified estimation of interaction. The interacting relationships were reported only through the observed differences in effect sizes across strata, which may result in an unconvincing conclusion of interaction estimation due to the confounding factors or low statistical power in some stratum [3, 8, 10].

For instance, in a meta-analysis reported by Meng F et al. [6], the nonsmoking population of two independent studies with 40 renal cell carcinoma (RCC) patients with 70 controls and 35 RCC patients with 135 controls was added to the anther independent size of 75 nonsmoking RCC patients and 205 nonsmoking controls [6] (Table 2). Then, these cases and controls were applied for the computation of the summary odds ratios (ORs) without considering their differences in origins. Second, in a meta-analysis by Nagao M et al. [4], the researchers calculated four pooled ORs for estimation (Table 3) and concluded that nonsteroidal anti-inflammatory drug (NSAID) use was associated with a decreasing cancer risk among both peroxisome proliferator-activated receptor gamma (*PPARγ*) rs1801282 CC carriers (*P* = 0) and CG + GG carriers (*P* = 0.006), whereas the *PPARγ* rs1801282 polymorphism (CG + GG vs CC) had little influence on developing cancer among non-NSAID users (*P* = 0.865) or NSAID users (*P* = 0.658) [4]. Although this approach enabled confounder control within a stratum, the effect of either the *PPARγ* rs1801282 polymorphism or NSAID intake failed to be revealed at an overall study population level. On the other hand, He W et al. [3] investigated the association between the murine double minute 2 (*MDM2*) rs2279744 T/G polymorphism and the risk of lung cancer by smoking status (Table 4) and showed that the *MDM2* rs2279744 polymorphism increases the risk of lung cancer among nonsmokers (TG vs. TT: *P* = 0.008; GG vs. TT: *P* < 0.001; GG + TG vs. TT: *P* = 0.001) but not among smokers (TG vs. TT: *P* = 0.896; GG vs. TT: *P* = 0.353; GG + TG vs. TT: *P* = 0.607), without considering the confounding caused by the stronger effect of tobacco smoking and applying a quantitative test to this observed “interaction”.

**Table 2:** Meta-analysis of the association of the CYP1A1 MspI polymorphism and smoking with the risk of renal cell carcinoma

Meta-analysis	No. of studies	CYP1A1 MspI	Non-smokers					Smokers				
			Cases	Controls	OR	95%CI	*P*	Cases	Controls	OR	95%CI	*P*
*Meng F* et al.*, 2015* [6]	2	Wt/Wt	75	205	1 (reference)		NA	67	128	1.43	0.96–2.13	0.08
		Wt/Vt + Vt/Vt	92	119	2.11	1.44–3.09	< 0.001	90	73	3.37	2.24–5.06	< 0.001

Note: *OR* odds ratio, *CI* confidence interval, *NA* not available, *CYP1A1* cytochrome P450 1A1

**Table 3:** Meta-analysis of the association of the PPARγ rs1801282 polymorphism and NSAID use with the risk of cancer

Meta-analysis	No. of studies	Genetic comparison	Non-NSAID users					NSAID users					
			*P*_*h*_	Model	OR	95%CI	*P*_*OR*_	*P*_*h*_	Model	OR	95%CI		*P*_*OR*_
*Nagao M* et al.*, 2014*	6	**CC**	NA		1 (reference)		NA	NA		1 (reference)			NA
		**CG + GG**	0.865	F	0.932	0.830-1.046	0.865	0.034	R	0.942	0.724-1.226		0.658
			**CC**					**CG + GG**					
		**Non-NSAID user**	NA		1 (reference)		NA	NA		1 (reference)			NA
		**NSAID user**	0.942	F	0.743	0.673-0.820	0	0.065	F	0.786	0.663-0.932		0.006

Note: *OR* odds ratio, *CI* confidence interval; *F* fixed-effect model; *NA* not available, *NSAID* nonsteroidal anti-inflammatory drug, *PPARγ* peroxisome proliferator-activated receptor gamma

**Table 4:** Meta-analysis of the MDM2 rs2279744 polymorphism in lung cancer by smoking status

Meta-analysis	No. of studies	Sources of results	Genetic comparison	Non-smokers						Smokers					
				*I*^*2*^	Model	OR	95%CI		*P*	*I*^*2*^	Model	OR	95%CI		*P*
*He W* et al.*, 2012* [3]	5	**A**	**TG vs. TT**	28.60%	F	1.275	1.066-1.526		0.008	72.40%	R	1.015	0.816-1.262		0.896
			**GG vs. TT**	0.00%	F	1.583	1.257-1.994		< 0.001	84.00%	R	1.201	0.816-1.768		0.353
			**GG + TG vs. TT**	40.50%	F	1.334	1.125-1.581		0.001	83.2%^a^	R	1.072	0.823-1.394		0.607
				**Non-smokers**						**Smokers**					
				*I*^*2*^	Model	OR	95%CI			*I*^*2*^	Model	OR	95%CI		
		**B**	**TG vs. TT**	–		1.271	1.063-1.521			–		1.013	0.846-1.212		
			**GG vs. TT**	–		1.521	1.214-1.905					1.200	0.886-1.625		
			**GG + TG vs. TT**	40.50%	F	1.328	1.119-1.575			–		NA	NA		

Note: *OR* odds ratio, *CI* confidence interval, *F* fixed-effect model, *NA* not available A: statistical results calculated by us using STATA 14.0; B: statistical results calculated by original authors using METAGEN; *MDM2* murine double minute-2

^a^ complementally calculated by us

To solve the issues described above, we discuss the logic of effect model selection in a stratification analysis and then provide systematic descriptions of the methodology, application and interpretation of three calculation methods for performing stratification analyses in a meta-analysis of molecular association, herein referred to as *factorial stratification analysis*, *confounder-controlling stratification analysis* and *standard stratification analysis*, on the basis of the original methods [2–4, 6–10] and complementary analyses conducted by us [5].

## Methods

### Effect model selection in a meta-analytic stratification analysis

The selection of the effect model is a matter of cardinal significance in a meta-analysis. Besides directly affecting the computing process, the model also serves the purpose to analysis and the interpretation of statistical results [15]. The pooled effect *M* is calculated as the weighted average of each included study effect size:

1 $$ \mathrm{M}=\frac{{\sum \limits}_{i=1}^k{W}_i{Y}_i}{{\sum \limits}_{i=1}^k{W}_i} $$

and the variance of the pooled effect is computed as:

2 $$ {V}_M=\frac{1}{\sum_{i=1}^k{W}_i} $$

where *W*_*i*_ is the weighting factor of study i and *Y*_*i*_ is the effect size for study i. Generally, two widely applied statistical models exist in meta-analysis: the fixed-effect model (FM) and the random-effects model (RM) [16]. In the FM, the weight of each part can be described as follows:

3 $$ {W}_i=\frac{1}{V_i} $$

where *V*_*i*_ is the variance of the mean for study i. Under the FM, we suppose that only one true effect size is shared by all included studies and that sampling errors lead to all observed variances in the analysis. In contrast, the weight assigned to each study in the RM is:

4 $$ {W}_i=\frac{1}{V_i+{T}^2} $$

where *V*_*i*_ and *T*^*2*^ are the estimated within-study variance and between-study variance, respectively. In the RM, we allow the true effect sizes to differ from study to study. The study previously conducted by us showed that suitably employing effect models could effectively resolve some abnormal phenomena occurring during a stratification analysis [5]. In a meta-analytic stratification analysis, two aspects should be taken into consideration during the effect model selection: (a) which effect model is appropriate for computing the effect within each stratum and (b) which effect model is appropriate for pooling the summary effects across strata. These two issues will be addressed separately as follows.

(I) *Within strata*: The criterion for the selection of an appropriate effect model in this step is the same as it was for the simple mate-analysis, except that the unit of analysis is a “substudy” (study included in a stratum) rather than a “study”. As generally recognized, FM or RM is selected according to the results of the test of homogeneity [17]. When the *P* value of the homogeneity test is more than 0.05 (sometimes 0.10 is defined as the test level for its low statistical power) or the *I*^*2*^ statistic of the χ^2^-based Q test is less than 50% [16], the multiple included substudies will be considered as having homogeneity, sharing a common effect across studies; then, an FM will be selected to synthesize the ORs. Otherwise, when the heterogeneity of variations was observed at a significant level, revealing that the true effects are different from one study to another, an RM will be chosen to estimate the mean effect of all included substudies.
(II) *Across strata*: After the effect sizes within strata are determined, we can proceed to compare these effect sizes and/or combine them to determine an overall estimate. For this purpose, we select an FM to complete this work. The reasons are as follows: (a) A test of homogeneity is conducted to examine the variation across strata, and the overall estimate will not be performed until this test shows no significant variation across strata, which also suggests no effect modification or interaction between the investigated factor and stratified variable [18, 19]. (b) The FM assumes that these multiple strata share a common true effect size and that the number of strata is finite and known, while the RM assumes that effect sizes vary by variants of characteristics across stratum and that the number of strata is infinite [18]. When we are working across strata, the assumption of the FM rather than the RM meets the results of the homogeneity test and serves our purpose.

In summary, when performing a meta-analytic stratification analysis, we recommend selecting the effect model of FM or RM based on a homogeneity test within stratum and employing the FM for estimation across strata.

### Factorial stratification analysis

The first type of stratification analysis we addressed here is a “factorial stratification analysis”, referring to one of the subtypes of stratification analysis in the previous meta-studies [2, 6, 20]. In this method, the effect sizes are calculated at different exposure levels to obtain a primary understanding of the roles of the investigated genetic factors, third-party variables and both on the risk of diseases. In detail, subjects with no genetic susceptibility in the unexposed stratum (e.g., nonsmokers carrying the wild-type allele or genotype) are regarded as a reference and compared with individuals with genetic susceptibility in the same stratum (e.g., nonsmokers carrying the mutant-type allele or genotype), subjects with no genetic susceptibility (e.g., smokers carrying the wild-type allele or genotype) and susceptible individuals (e.g., smokers carrying the mutant-type allele or genotype) in the exposed stratum, which can be clearly understood in Table 5A.

(I) In the three pairwise comparisons mentioned above, we can compute ORs for all other groups and evaluate between-study heterogeneity according to Cochran’s χ^2^-based Q and *I*^*2*^ statistic tests [16].
(II) Appropriate meta-analysis models are selected to compute the pooled ORs, including OR_2−_, OR_1+_ and OR_2+_) with corresponding 95% confidence intervals (CIs) for different comparisons based on a heterogeneity test. As mentioned above, the selection of the effect model within stratum should be based on the results of the test of homogeneity [17]. Here, if *I*^*2*^ < 50%, the FM (the Mantel-Haenszel (M-H) method) [1] is applied. Otherwise, we will select the RM (the Der Simonian-Laird (D-L) method) [21].
(III) On the basis of the above steps and referring to the methodology on a single-study level [22], we can suggest additionally calculating another two effect sizes for the comprehensive estimation of whether an interacting relationship exists between the investigated genetic factor and stratified moderator and further quantifying the strength of this interaction at a meta-analytic level. The interaction contrast ratio (ICR) [23] with a 95% CI obtained by the delta method [24] was computed for the additive scale:

**Table 5:** Factorial stratification analysis and confounder-controlling stratification analysis

**A.**			
Exposure	Stratum 1	Stratum 2	ICR and OR_int_
–	Reference	OR_2−_	$$ {\displaystyle \begin{array}{c} ICR={OR}_{2+}-{OR}_{1+}-{OR}_{2-}+1\\ {}{OR}_{int}={OR}_{2+}/\left({OR}_{1+}\times {OR}_{2-}\right)\end{array}} $$
+	OR_1+_	OR_2+_	
**B.**			
Exposure	Stratum 1	Stratum 2	Combined OR
–	Reference1	Reference2	${OR}_{IV}=\frac{W_{IV1}{OR}_1+{W}_{IV2}{OR}_2}{W_{IV1}+{W}_{IV2}}$
+	OR_1_	OR_2_	

Note: *OR* odds ratio, *ICR* interaction contrast ratio

5 $$ ICR={OR}_{2+}-{OR}_{1+}-{OR}_{2-}+1 $$

and the OR_int_ with a 95% CI was computed for the multiplicative scale [22]:

6 $$ {OR}_{int}=\frac{OR_{2+}}{OR_{1+}\times {OR}_{2-}} $$

where *OR*_*1+*_, O*R*_*2−*_, and *OR*_*2+*_ are the effect sizes calculated in Step (II). The ICR (or OR_int_) reveals the relative strength of the observed effect compared to the theoretically superposed effect of the two factors at a meta-analytic level by an additive model (or the multiplicative model). When two factors share homogenous effects on outcome, the larger or smaller the ICR deviating from 0 is (or OR_int_ deviating from 1), the stronger the interacting effect between the investigated factor and stratified moderator is. Instead, when their effects are opposite, ICR > 0 (or OR_int_ > 1) suggests that the interaction increases the risk of developing disease, while ICR < 0 (or OR_int_ < 1) suggests that the interacting effect plays a protective role in disorder. Ideally, the ICR (or OR_int_) should be 0 (or 1) if the protective and destructive effects, respectively, from these two factors can be neutralized. Notably, this step is usually not included in the former practical method [2, 5, 6, 20] to perform this kind of stratification analysis, as reported in previous studies.

As shown in Table 5A, the role of each factor is independently assessed for both individual and joint effects on the risk of disease, and in addition, the ORs can be examined in terms of departure from specified models of independence (the additive model or the multiplicative model) [25], so we call this stratification analysis a “factorial” method.

### Confounder-controlling stratification analysis

In a primary case-control study, another classical subtype of stratification analysis was used to control confounding factors and reveal the real relationship of genetic susceptibility with disease by computing the effect sizes within the stratum using an M-H or inverse-variance (I-V) approach used to combine them [1]. In published meta-analyses of genetic associations at a population level, an analogous subtype of stratification analysis was also generally performed to process stratified data [3–5, 7–9], in which the ORs are pooled within the same stratum but not combined across strata. The advanced subtype can be visually understood, as shown in Table 5B.

(I) Pooling the stratified data of each included study within every stratum with the FM (the M-H method) or the RM (the D-L method) based on the *I*^*2*^ statistic of the heterogeneity test. The algorithms of this substep are the same as those in *Step (I)* and *Step (II)* in the factorial stratification analysis and in the simple meta-analysis.
(II) Checking heterogeneity across strata. The χ^2^-based Q test is conducted to estimate the variation between the ORs with 95% CIs across strata. Further analysis of these strata will depend on whether the variation across strata is significant.
(III) If variation across strata does not show any statistical significance (the *P* value of χ^2^ statistic is greater than 0.10), indicating that the true effects of investigated factors across strata are identical, then the FM (the I-V method) is utilized to combine the effect sizes with the upper and lower CIs in each stratum:

7 $$ {OR}_{combined}=\frac{\sum_{i=1}^q\left({W}_{IVi}\times {OR}_i\right)}{\sum_{i=1}^q{W}_{IVi}} $$

where *OR*_*i*_ is the pooled effect size in each stratum and *w*_*IVi*_ is the weight assigned to each stratum using the I-V method. The theory of employing FM is based on three aspects of the abovementioned assumptions: (a) all strata share the same true effect; (b) this true effect is a point value; and (c) the number of strata is defined previously. These assumptions meet the situation when the test of homogeneity indicates no significant variation across strata.

(IV) The crude OR was computed using the overall sample size without stratification and then compared with the adjusted OR via stratification (OR_combined_). Any inconsistency in statistical significance between the effect sizes suggests that the stratified moderator serves as a confounding factor in the analysis.
(V) If the variation across strata is significant (the *P* value of χ^2^ statistic is less than or equal to 0.10), then the true effects of the investigated factors across strata are different. The stratification moderator will be considered an interacting or effect modification factor for the relationship between the genetic variant and the risk of disease. Interaction or effect modification is a constant, natural phenomenon and is not associated with the design of the study. The RM is not suitable for combining effect sizes across strata because it assumes that the number of strata is infinite, which is contrary to the actual situation.

As mentioned above, the main goal of this step is to control the confounding variables and reveal the real effect of the investigated factor; we call this analysis a “confounder-controlling stratification analysis”. Notably, four steps (II-VI) are usually not included in the former practical method [3–5, 7–10] to perform this kind of stratification analysis, which can also be called a subgroup-type stratification analysis, as reported in previous studies.

### Standard stratification analysis

**Fig. 2:**
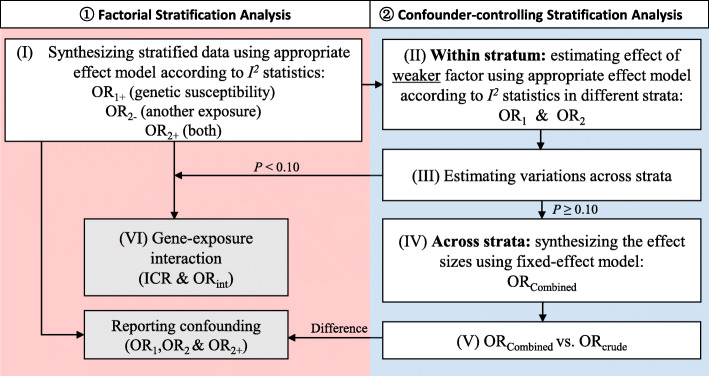
Flow diagram of the process of standard stratification analysis in meta-analyses

As discussed above, the former two subtypes of meta-analytic stratification analyses have their own advantages and disadvantages. On one hand, we can use a factorial stratification analysis to reveal when exposure or confounding variables have a multilevel viewpoint, particularly the double effect of two confounding or interacting factors in an overall study population, to ascertain whether the investigated genetic factor interacts with the stratification moderator and to further quantify the strength of the interacting effect. This method can effectively solve issue (c) described in the Introduction but not issue (b). On the other hand, a confounder-controlling stratification analysis can be used to identify the confounding or interacting variables via stratification and further uncover the true effects of genetic susceptibility among the overall study population. This variant of analysis can effectively resolve issue (b) described in the Introduction but not issue (c). Thus, a complete stratification analysis could include the above two methods to solve both issues. Therefore, we further provide a “standard stratification analysis” by supplying and extending the statistical algorithms of our previously established analytic approach [5], which can regard both factorial stratification and confounder-controlling stratification analyses as subtypes. A flow diagram of the standard stratification process in the meta-analysis is detailed in Fig. 2.

(I) Estimating the effects of genetic susceptibility and exposure by synthesizing stratified data using an appropriate effect model and computing the summary ORs of interest, OR_1+,_ OR_2−_ and OR_2+_ (same as Steps (I) to (II) of a factorial stratification analysis). The algorithms of this substep are the same as those in the former two types of stratification analysis.

(II) to (V) A confounder-controlling stratification analysis is performed to investigate the weaker effect between the two factors displayed in Step (I) first, and then the stronger effect is explored (OR_1+_ vs. OR_2−_). The real role of the weaker variable is more difficult to determine under the confounding impact of the stronger factor. The aim of this analysis is to control the potential confounding of the stronger effect and reveal the weaker effect.

(VI) If the variation across strata is significant (the *P* value of the χ^2^ statistic is less than or equal to 0.10), then the true effects of the investigated factors across strata are different. The stratification moderator will be considered an interacting factor for the relationship between the genetic variant and the risk of disease. The logic of this step is the same as that in the confounder-controlling stratification analysis. To further quantify the strength of the interacting effect, ICR and OR_int_ with 95% CIs were calculated to show the impact on the risk of the disease when both factors simultaneously exist. Algorithms of these steps also refer to those (Step (III)) used in the factorial stratification analysis.

**Fig. 3:**
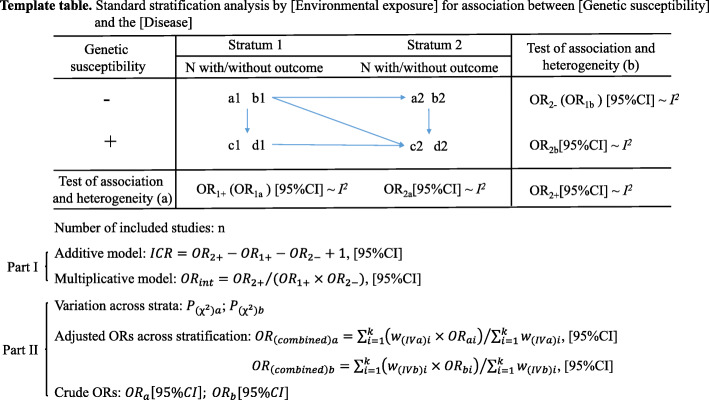
Template table of the standard stratification analysis in meta-analyses

The standard stratification analysis served as a combination of two subtypes of stratification methods, factorial stratification analysis and confounder-controlling stratification analysis, which included Steps (I) and (VI) and Steps (II) to (V), respectively. We propose a template table for presenting the statistical results of this analysis, which will allow readers to obtain the information needed to assess the association of interest (Fig. 3). Notably, a1, b1, c1, d1, a2, b2, c2 and d2 represent the sum of relative ones in the included studies, which helps to obtain a rough estimation of the distribution of the summary cases and controls in each item. We also provide step-by-step instructions in a table format to make it easier to follow for meta-research practitioners (Table S1). This method of analysis and data presentation not only reveals an overall effect of genetic variants by controlling confounders but also provides a quantified estimation of the strength of the interaction, giving us a comprehensive view of the relationship between the genetic factor and a third party as well as their effects on the disease outcome.

## Results

To illustrate this point in detail, a standard stratification analysis was performed using two examples of previous meta-analyses [3, 4] concerning molecular association in cancers developments, which were mentioned in the Introduction. STATA version 14.0 software (STATA Corporation, College Station, TX, USA) with the software package *metan* [26] was utilized to complete the analytic work in this paper.

### Example 1: The PPARγ polymorphism and NSAID usage in the cancer risk

The first meta-analysis used here for illustrative purposes investigated the roles of the *PPARγ* rs1801282 polymorphism and the intake of NSAID in developing cancer, which collected eight studies involving 4269 cases and 5903 controls [4], and all studies consisted of stratified data (Table S2). Two aims were proposed by the original researchers: (a) to investigate the connection between the *PPARγ* rs1801282 variant and the risk of cancer and (b) to determine the influence of NSAID usage on thwarting cancer. In this work, we conducted a standard stratification analysis to achieve these goals following the steps described below.

First, we applied a factorial stratification analysis to estimate the effect sizes given that the roles of both the *PPARγ* variant and NSAID intake were uncertain before our analysis. The unilateral effect of the *PPARγ* rs1801282 variant was not statistically significantly linked to the cancer risk (OR (95% CI) = 0.932 (0.830–1.046), *P* = 0.865), although NSAID usage unilaterally reduced this risk by a significant level (OR (95% CI) = 0.743 (0.673–0.820), *P* < 0.001) (Table 6). This result supported the original conclusion [4]. However, when considering both of the above effects, the OR did not show a significant association between the additive effect and the risk of cancer (OR (95% CI) = 0.765 (0.576–1.015), *P* = 0.064). These ORs implied that a potential effect of modification or interaction might occur between these two factors since the effect of NSAIDs was covered under the mutation of *PPARγ* polymorphism and their combined effect did not decrease the risk of developing cancer.

**Table 6:** Standard stratification analysis by NSAID use status for the association between the PPARγ rs1801282 polymorphism and the risk of cancer

PPARγ rs1801282	Non-NSAID users		NSAID users		Test of association and heterogeneity (b)
	Case	Control	Case	Control	
**CC**	2208	2773	1059	1653	0.743 (0.673–0.820) ~ 46.70%
**CG + GG**	664	902	338	575	0.811 (0.622–1.056) ~ 51.8%
**Test of association and heterogeneity (a)**	0.932 (0.830–1.046) ~ 0.00%		0.942 (0.724–1.226) ~ 58.70%		0.765 (0.576–1.015) ~ 68.30%

Number of included studies: 6

Variation across strata: ${P}_{\left({\upchi}^2\right)a}=0.942$; ${P}_{\left({\upchi}^2\right)b}=0.543$

Adjusted ORs across stratification: *OR*_(*combined*)*a*_(95 % CI) = 0.934 (0.840 − 1.038); *OR*_(*combined*)*b*_(95 % CI) = 0.751 (0.685 − 0.824)

Crude ORs: *OR*_*a*_(95 % CI) = 0.927 (0.845 − 1.017); *OR*_*b*_(95 % CI) = 0.799 (0.840 − 1.038)

In the above analysis, the unilateral effect of the *PPARγ* rs1801282 polymorphism was much weaker than that of NSAID intake. Thus, we first investigated the effect of this genetic polymorphism. In the following analysis, our aim is to provide an accurate assessment of the effect of this variant among the overall population but not the unilateral effect in the population with a particular drug intake status. Therefore, NSAID intake can serve as a potential confounder and its effect should be controlled. As shown in Table 6, the χ^2^-based Q test calculated for a confounder-controlling stratification analysis indicated no significant variation across strata (χ^2^ = 0.01, *P*_*(χ2)a*_ = 0.942). Therefore, we combined the effect sizes of these two strata, and the combined OR indicated that the *PPARγ* rs1801282 variant did not correlate with the risk of cancer at the overall level (OR (95% CI) = 0.934, (0.840–1.038)). Second, considering the potential effect of the modification or interaction between two exposure factors, we further investigated the effect of NSAID intake on the cancer risk and calculated the pooled ORs stratified by the carrying status of the *PPARγ* rs1801282 polymorphism (CC carriers: OR (95% CI) = 0.743 (0.673–0.820), *P* < 0.001; CG or GG carriers: OR (95% CI) = 0.811 (0.622–1.056), *P* = 0.12, shown in Table 6). According to our statistical results, NSAID usage was associated with a decreased risk of cancer among *PPARγ* rs1801282 wild-type homozygous (CC) individuals but not among mutant-type allele carriers (CG or GG). Then, the χ^2^-based Q test demonstrated that effect sizes did not change significantly across strata (χ^2^ = 0.37, *P*_*(χ2)b*_ = 0.543), therefore, we also combined the effect sizes of these two strata, and the combined OR demonstrated that NSAID intake can significantly decrease the risk of cancer among the overall study population (OR (95% CI) = 0.751 (0.685–0.824), as shown in Table 6).

The comparison between the results of our current method and the original method is presented in Table S3. It is worth noting that in the original paper, the authors used the FM rather than the RM under the between-study heterogeneity of *P*_*h*_ = 0.065 and *I*^*2*^ = 51.8% and obtained the different results from ours: CG or GG carriers: OR (95% CI) = 0.786 (0.663–0.932), *P* = 0.006 (Table 3 and Table S3), possibly for the consistency of the NSAID use effect. Nevertheless, we obtained the results by employing the RM to complete this work under the same situation (OR (95% CI) = 0.811 (0.622–1.056), *P* = 0.12, shown in Table 6). This phenomenon implies that the conclusions within stratum are somewhat “model-dependent”. In other words, the results are often different or difficult to interpret due to diversity in the model selection criteria. On the other hand, compared to the approach of reporting effects within strata alone, the approach of testing homogeneity and pooling effect sizes across strata is more reliable and robust to assess the gene-disease or exposure-disease associations.

In contrast, we also calculated the crude ORs for the meta-analysis of the unilateral effects of the *PPARγ* rs1801282 polymorphism or NSAID intake (*PPARγ* rs1801282 polymorphism: OR (95% CI) = 0.927 (0.845–1.017), *P* = 0.11; NSAID use: OR (95% CI) = 0.799 (0.840–1.038), *P* = 0.204), which did not show any association of either of these two variables with cancer risk. This result generated the same conclusion with either the effect sizes of the *PPARγ* rs1801282 polymorphism within each stratum or among the overall population but not with those of NSAID use. The difference from the crude OR and adjusted OR of NSAID use can be due to the residual confounding that caused wider CIs and larger *P* values.

In sum, the standard stratification analysis (a) determined the true effects of both the *PPARγ* rs1801282 variant and NSAID usage on the risk of cancer in the overall study population after controlling for confounding factors and (b) clarified an unconventionally negative result of NSAID use among mutant-type carriers, which could be easily obtained but would be difficult to explain in the conventional stratified analysis.

### Example 2: MDM2 polymorphism and smoking status in the development of lung cancer

Numerous studies have previously demonstrated that cigarette smoking represents a widely recognized risk factor for lung cancer [27, 28]. Therefore, when He W et al. [3] investigated the association between the *MDM2* polymorphism and the development of lung cancer, the effect of cigarette smoking was considered because different distributions of smoking individuals between strata might represent a confounding variable.

In this meta-analysis, the authors collected the stratified data for the *MDM2* rs2279744 polymorphism by smoking status from five of the nine included studies (Table S2) and calculated the pooled OR of the homozygous wild-type TT compared with the TG and GG genotypes of *MDM2* rs2279744 among smokers, which suggested no association between this genetic polymorphism and the lung cancer risk among smokers (Table 4) [3]. In contrast, we complementally calculated the pooled OR under the dominant model (OR (95% CI) = 1.072 (0.823–1.394), *P* = 0.607, shown in Table 4), which supported the result of the genetic model selection in the original paper. On the other hand, the *MDM2* rs2279744 polymorphism showed significant associations with the risk of lung cancer among nonsmokers (OR (95% CI) = 1.334 (1.125–1.581), *P* = 0.001). However, the limitations of this retrospective work should be recognized: (a) in the original paper, the researchers assumed that the *MDM2* rs2279744 polymorphism increases the risk of lung cancer among nonsmokers but not among smokers [3], without considering the false interaction due to the confounding caused by the stronger effect of tobacco smoking; and (b) the original study was not able to control for this confounding variable and uncover the real effect of the *MDM2* rs2279744 polymorphism at the level of the overall population.

In the current study, we conducted a standard stratification analysis to resolve the above issues. The statistical results by our calculation are shown in Table 7, and the merged table comparing the original reported result side by side with the new results is presented in Table S4. In the factorial stratification analysis, the *MDM2* rs2279744 polymorphism was statistically significantly associated with lung cancer among the nonsmokers (OR (95% CI) = 1.334 (1.125–1.581), *P* = 0.001). Additionally, cigarette smoking had a nearly 1.70-times greater effect on increasing the risk of developing lung cancer (OR (95% CI) = 2.274 (1.015–5.094), *P* < 0.046), compared with the *MDM2* rs2279744 polymorphism. Under the double effect of the *MDM2* rs2279744 polymorphism and cigarette smoking, the OR increased contrasted with that for smoking only, thus suggesting an enhanced risk of lung cancer (OR (95% CI) = 2.469 (1.116–5.461), *P* = 0.026). The factorial stratification analysis provided more information than thethe original subgroup-type stratification analysis in that the former offered insights into the influence of cigarette smoking and its additive effect with the *MDM2* rs2279744 polymorphism on the risk of lung cancer (Table S4).

**Table 7:** Standard stratification analysis by smoking status for the association between the MDM2 rs2279744 polymorphism and the risk of lung cancer

MDM2 rs2279744	Non-smokers		Smokers		Test of association and heterogeneity (b)
	Case	Control	Case	Control	
**TT**	273	861	1393	1208	2.274 (1.015–5.094) ~ 95.50%
**TG + GG**	711	1057	2363	2253	1.796 (0.735–4.387) ~ 98.30%
**Test of association and heterogeneity (a)**	1.334 (1.125–1.581) ~ 40.50%		1.072 (0.823–1.394) ~ 83.20%		2.469 (1.116–5.461) ~ 95.90%

Number of included studies: 5

Variation across strata: ${P}_{\left({\upchi}^2\right)a}=0.160$; ${P}_{\left({\upchi}^2\right)b}=0.701$

Adjusted OR across stratification: OR_(combined)a_(95 % CI) = 1.232 (1.054 − 1.410); OR_(combined)b_(95 % CI) = 2.045 (1.124 − 3.722)

Crude ORs: *OR*_*a*_(95 % CI) = 1.110 (0.871 − 1.414); *OR*_*b*_(95 % CI) = 1.908 (0.793 − 4.589)

As previously shown, the *MDM2* rs2279744 polymorphism showed a much weaker effect than smoking. We studied the effect of the *MDM2* rs2279744 polymorphism and controlled for cigarette smoking as a potential confounder in the analysis. In the following confounder-controlling stratification analysis, no significant variations were observed across strata in the examination of the difference between the two ORs (χ^2^ = 1.98, *P*_*(χ2)a*_ = 0.160); therefore, an FM was utilized to synthesize these two ORs (shown in Table 7). The combined OR indicated that the *MDM2* rs2279744 polymorphism also correlated with an increasing risk of lung cancer in the overall population at a significant level (OR (95% CI) = 1.232 (1.054–1.410)). However, the crude OR failed to display that the correlation was statistically significant (OR (95% CI) = 1.110 (0.871–1.414), *P* = 0.401).

However, if we want to verify the effect of smoking on lung cancer risk, the carrier status of the *MDM2* rs2279744 polymorphism should be regarded as a stratified moderator and then controlled as well. Among individuals with the TT genotype, smoking increased the risk of lung cancer (OR (95% CI) = 2.274 (1.015–5.094), *P* = 0.046), while smoking showed no association with this risk in TG or GG carriers (OR (95% CI) = 1.796 (0.735–4.387), *P* = 0.199). The results from the homogeneity test across strata suggested no significant variation (χ^2^ = 0.15, *P*_*(χ2)b*_ = 0.701). Therefore, the FM (I-V method) was employed to combine the across-strata effect sizes. The combined OR indicated that smoking is a risk factor for lung cancer among the overall study population (OR (95% CI) = 2.045 (1.124–3.722)), which is consistent with the conclusions of numerous previous studies [27, 28]. However, the crude OR without stratification showed no significant association between smoking and lung cancer (OR (95% CI) = 1.908 (0.793–4.589), *P* = 0.149) and failed to reveal this relationship under the confounding of the *MDM2* rs2279744 polymorphism, further confirming the importance of the standard stratification analysis in controlling confounder effects and revealing the real relationship between exposure and outcome.

## Discussion

In this paper, we provide a systemic study of stratification analyses in ameta-analysis of molecular associations. Based on two previous approaches [2–4, 6–10, 20], we propose a methodology of stratification analyses and demonstrate its application in three types of meta-analyses: *factorial stratification analysis*, *confounder-controlling stratification analysis* and *standard stratification analysis*. The third method synthesizes the advantages of the first two methods, so two examples have been used for illustration.

In these two examples, an interesting result occurred when we studied the influence of NSAID intake on the cancer risk in the first meta-analysis and the effect of the *MDM2* rs2279744 polymorphism or smoking on susceptibility to lung cancer in the second meta-analysis. The adjusted ORs by stratification suggested that these investigated factors decreased or increased the risk of diseases at a significant level, but the crude OR showed no effect. We consider that the adjusted ORs are more reliable than the crude ORs, which clarify the true association among NSAID use, the *MDM2* rs2279744 polymorphism, smoking and the risk of diseases, because synthesizing the effect sizes according to the heterogeneity first within each stratum and then across strata (“two-step” approach) offers better results than utilizing the overall between-study heterogeneity (“one-step” approach) in the meta-analysis. The statistical difference between these two ORs indicated a residual confounding in the first example, and smoking status was a strong confounder in the second example. On the other hand, the results from the overall population (OR_combined_) had stronger statistical power due to its larger sample size than that in the unilateral analysis of NSAID use among individuals carrying particular *PPARγ* rs1801282 alleles or the *MDM2* rs2279744 polymorphism in individuals who never smoked in the previous meta-analyses. Based on the given cases, the standard methodology for stratification analyses has exhibited its importance in exploring and controlling confounding variables in meta-research.

### Nomenclature of strata and subgroups

Subgroup analysis is another subtype of meta-analysis for detecting the source of between-study heterogeneity and comparing effects among different groups on an outcome [17, 19]. In a subgroup analysis, an included study is regarded as a unit and allocated to one group [19], whereas in the stratification analysis that we address here, an included study is separated into several parts, which can be called “substudies”, and substudies with identical characteristics are in the same strata. From this point of view, most of the “subgroup analyses” applied in the previous meta-analysis can be regarded as the special case of the stratification analysis, in which the included studies were of all types, such as “Study 4” in Fig. 1 (the characteristic has several levels while each included study only represents one of these levels, e.g., when the subgroup moderator is ethnicity, the included studies of association between dopamine beta-hydroxylase polymorphisms and neurodegenerative diseases were divided into 25 subgroups involving Caucasian subjects and 16 subgroups involving Asian subjects [29]). However, it is worth noting that this type of data could be used for conducting only the confounder-controlling analysis but not the factorial stratification analysis or the standard stratification analysis because the ORs of interest (OR_2−_, OR_1+_ and OR_2+_) cannot be calculated.

### Additional approaches to process stratified or subgroup data in meta-analyses

The issue in statistical analysis is that only a small proportion of included studies reported the stratified data or the effect sizes of different strata or subgroups in the meta-analysis. For example, in a meta-analysis evaluating the association between erectile dysfunction (ED) and the risk of cardiovascular disease (CVD), one of the twelve included studies reported individual hazard ratios for reduced erectile rigidity and severely reduced erectile rigidity, but the other studies did not [11]. Additionally, two of the twelve original studies reported subgroups of vegetable consumption (e.g., yellow, green and other) included in a meta-analysis of the association of vegetable intake with the gastric cancer risk [13]. Facing these issues, the standard stratification analysis might not be suitable to address confounding or interacting factors. However, we may ask whether it is useful to combine the original stratified data or the effect sizes of different strata and how. Three different methods for addressing this problem were found in previous meta-studies:

(I) Each stratum within an original study is treated as a “independent study” and then added to the effect model to synthesize the pooled effect size [30];
(II) Each stratum within an original study is combined into a summary effect size using an FM or an RM and then these effect sizes are pooled again with those of other studies for the main meta-analysis (e.g., use an FM [11, 12] and use an RM [13, 14]);
(III) For meta-analyses including original studies reporting stratified data rather than the effect sizes in each strata only, some researchers will add them directly and calculated effect size, and then pool these effect size with the other studies under an effect model for the main meta-analysis [2, 6–8, 20].

The logic to used process such rare stratified or subgroup data is the same as that for the model selection discussed above. Among these three methods, Method (III) was the most commonly misused in previous meta-analyses. Our previous study showed that this method may cause aberrant results or lead to incorrect conclusions due to different effects across strata [5]. For Method (II), although the between-substudy and between-study variance is considered, the relationship across strata is not determined clearly in this way. For considering the variance across strata within an original study, due to the confounding or interacting effect, or even other reasons, such as the sample error, the homogeneity test should be conducted. When the test shows no significant heterogeneity, an FM should be chosen to synthesize the stratified data, then the effect size would represent the homogeneity in this study and could then be used for pooled estimation across studies in a meta-analysis. Otherwise, the test suggests that there is a possible interaction, and these strata should not be combined for the representative effect size of their original studies. However, for the overall estimation of the effect of the investigated factor, Method (I) can be selected to complete this work under some uncertainty. Simultaneously, interacting relationships should be reported, and other methods, such as sensitivity analysis, should be used to explore changes in the overall heterogeneity among studies, alleviate the uncertainty caused by the interaction or other reasons, and examine the robustness of the results.

### Scopes of application of the standard stratification analysis and its two subtype analyses

The application scopes of standard stratification analysis, factorial stratification analysis and confounder-controlling stratification analysis should be recognized in meta-research. On the one hand, the standard stratification analysis is comprehensive and powerful. However, in order for this method to be used, the stratified data from original studies should be sufficient, or the method will not be appropriate for elucidating confounding or interacting effects.

On the other hand, when stratified data are rare in the included studies and/or there is no occurrence of abnormal results, we may simply use the two subtype analyses rather than the standard stratification analysis. Whether the factorial stratification analysis or the confounder-controlling stratification analysis should be used depends on the purpose of the stratified analysis. The former method aims to elucidate the roles of exposure or confounding factors and their additive effects and thus help to provide a multilevel perspective of the gene-disease association. This analysis can be performed separately when the sample size within each comparison is large enough. To our knowledge, numerous studies have adopted this practice [2, 6, 20]. Additionally, the ICR and OR_int_ can also be calculated to estimate the interaction strength. The latter method aims to reveal the true effects of exposure factors or to determine whether interacting relationships exist between exposure and confounding variables. This analysis can be conducted separately to control for confounders when the effect of the stratified moderator is clear and the test of homogeneity across strata indicates no interaction. Many meta-analysis studies have conducted this kind of analysis but did not employ a test of homogeneity or combine the effect sizes across strata [3, 4, 7–10]. The procedure for selecting among these three approaches is not absolutely standardized; instead, it can be adjusted by the authors as required for the specific analysis at hand.

### Further applications of stratification analyses

In the current paradigm of genetic epidemiology, a single complicated disease is considered to be associated with multiple pathways, multiple genes and multiple polymorphisms. Each single locus frequently has a significant but small effect on the occurrence and development of disease [31]. As a consequence, such a small effect will require large sample sizes to detect its impact and is more easily covered up by stronger factors. It is difficult to control such confounding through individual matching. Therefore, the application of stratification analyses will effectively improve the utilization of published data and enlarge the study sample sizes in meta-studies.

Additionally, although these stratification analyses were applied only for meta-analyses of observational studies with categorical outcomes here, this method can refer to meta-studies with other methodological designs, such as experimentally designed studies or those including contiguous data (e.g., the meta-analysis on the association of neprilysin mRNA levels and enzyme activity with risk of AD [32], or MGMT promoter methylation levels risk with ovarian cancer [33]). In such studies, the effect sizes of the standardized mean difference (SMD) or weighted mean difference (WMD) instead of the OR or risk ratio (RR) will be selected for estimating the strength of association, but the logic of the arithmetic is the same.

Moreover, as is commonly considered, the occurrence and development of complicated diseases are not only regulated by genomic or epigenomic variations but also influenced by environmental factors. These three types of stratification analyses also offer advantages for discovering the potential gene-gene, gene-environment or gene-drug interactions or effect modification by a simple approach and provide follow-up studies with more perspectives. This approach can also be a methodological supplement to other methods of meta-analysis besides meta-regression [34] and logistic regression [35].

In summary, with the rising number of genome-wide association studies (GWASs) or epigenome-wide association studies (EWASs) conducted, stratification analyses will be helpful for controlling confounding factors and for further exploration of the influences of their modifications on the occurrence of complex diseases at a multivariate level.

## Conclusion

Our study systematically presented the statistical methodology, theoretic algorithm, computing processes and applications of stratification analyses for meta-analyses. The major contributions of this paper include the following: (a) describing the computing processes and applications of three types of stratification analyses in meta-analysis, including factorial stratification analysis, confounder-controlling stratification analysis and standard stratification analysis; (b) establishing a detailed statistical algorithm and an interpretation of this method; (c) providing a template table for presenting the statistical results of the standard stratification analysis; and (d) discussing and resolving other methods for managing stratified data that are frequently utilized in previous meta-research. The two cases shown in this study provide a good perception of the methodology for standard stratification analyses, and these examples also indicate that this method plays an important role in confounder control when studying the associations of genetic polymorphisms with the risks of diseases. More multicenter studies designed to resolve gene-environment, gene-drug and gene-gene interactions or modifications are required to validate this method in the future.

## Supplementary information

**Additional file 1: Table S1.** Step-by-step instructions for standard stratification analysis in table format. **Table S2.** Stratified data of the included studies in two meta-analyses for illustration. **Table S3.** Comparison between stratification analysis in original meta-analysis by Nagao M. et al. and standard stratification analysis in current research. **Table S4.** Comparison between stratification analysis in original meta-analysis by He W. et al. and standard stratification analysis in current research

## Data Availability

The datasets used and/or analyzed during the current study are available in the *SUPPLEMENTARY MATERIAL*.

## Abbreviations

RCC: Renal cell carcinoma
OR: Odds ratio
NSAID: Nonsteroidal anti-inflammatory drug
*PPARγ*: Peroxisome proliferator-activated receptor gamma
*MDM2*: Murine double minute 2
FM: Fixed-effect model
RM: Random-effects model
ICR: Interaction contrast ratio
CI: Confidence interval
M-H method: Mantel-Haenszel method
D-L method: Der Simonian-Laird method
I-V method: Inverse-variance method
ED: Erectile dysfunction
CVD: Cardiovascular disease
SMD: Standardized mean difference
WMD: Weighted mean difference
RR: Risk ratio
GWASs: Genome-wide association studies
EWASs: Epigenome-wide association studies
