# Supplementary material for: Advanced stratification analyses in molecular association meta-analysis: methodology and application
Source: BMC Med Res Methodol. 2020 Jun 8;20:147. doi: 10.1186/s12874-020-01020-z (PMC7278161; doi:10.1186/s12874-020-01020-z)
Supplement: Supplementary file 1 — Additional file 1: Table S1. Step-by-step instructions for standard stratification analysis in table format. Table S2. Stratified data of the included studies in two meta-analyses for illustration. Table S3. Comparison between stratification analysis in original meta-analysis by Nagao M. et al. and standard stratification analysis in current research. Table S4. Comparison between stratification analysis in original meta-analysis by He W. et al. and standard stratification analysis in current research [file 12874_2020_1020_MOESM1_ESM.docx]

**SUPPLEMENTARY MATERIAL**

**Table S1**  Step-by-step instructions for standard stratification analysis in table format

| **Standard stratification analysis** | | |
| --- | --- | --- |
| **Step** | **Process** | **Subtype Analysis** |
| (I) | - Calculating ORs of interest (OR_1+,_ OR_2-_ and OR_2+_)   • Checking heterogeneity (within comparison)  • Synthesizing data using appropriate effect model (FM or RM) | Factorial stratification analysis I |
| (II) | - Estimating weaker factor by stratification   • Choosing to investigate weaker factor (OR_1+_ vs. OR_2-_)  • Checking heterogeneity (within stratum)  • Synthesizing data using appropriate effect model (FM or RM)  • Calculating OR_1a_ and OR_2a_ (or OR_1b_ and OR_2b_) | Confounder-controlling stratification analysis |
| (III) | - Checking heterogeneity (across strata)   - If P ≥ 0.10, go to Step (IV)  - If P < 0.10, go to Step (VI) |  |
| (IV) | - Calculating adjusted OR   • Combining OR_1a_ and OR_2a_ (or OR_1b_ and OR_2b_) |  |
| (V) | - Detecting confounding   • Calculating crude OR  • Comparing adjusted OR and crude OR  - If contradiction exists, report confounding  - If no contradiction exists, no confounding detected |  |
| (VI) | - Assessing interaction   • Calculating ICR and OR_int_ | Factorial stratification analysis II |

**Table S2**  Stratified data of the included studies in two meta-analyses for illustration

Note: PPARγ: peroxisome proliferator-activated receptor gamma; NSAID: nonsteroidal anti-inflammatory drug; MDM2: murine double minute-2; *Stratum 1: Nago M et al., 2014: non-NSAID users; He W et al., 2012: nonsmokers; Stratum 2: Nago M et al., 2014: NSAID users; He W et al., 2012: smokers.

**Table S3** Comparison between stratification analysis in original meta-analysis by Nagao M. et al. and standard stratification analysis in current research

Note: Bold: results of stratification analysis from original meta-analysis by Nagao M. et al..

*This result was obtained using fixed-effect model.

**Table S4** Comparison between stratification analysis in original meta-analysis by He W. et al. and standard stratification analysis in current research

 Note: Bold: results of stratification analysis from original meta-analysis by He W. et al. using METAGEN.
